# Systemic therapy for laryngeal carcinoma

**DOI:** 10.3389/fonc.2025.1541385

**Published:** 2025-03-04

**Authors:** Thorsten Fuereder, Florian Kocher, Jan Baptist Vermorken

**Affiliations:** ^1^ Department of Medicine I, Medical University of Vienna, Vienna, Austria; ^2^ Department of Internal Medicine V (Hematology and Oncology), Medical University of Innsbruck, Innsbruck, Austria; ^3^ Faculty of Medicine and Health Sciences, University of Antwerp, Antwerp, Belgium; ^4^ Department of Medical Oncology, Antwerp University Hospital, Edegem, Belgium

**Keywords:** laryngeal squamous cell carcinoma, head and neck squamous cell carcinoma, immunotherapy, larynx preservation, neoadjuvant

## Abstract

Laryngeal squamous cell carcinoma (LSCC) accounts for 100,000 deaths worldwide each year. Despite multimodal treatment, outcomes for both high-risk locally advanced and recurrent/metastatic laryngeal carcinoma remain poor. Treatment intensification through induction chemotherapy has not improved overall survival, although it may contribute to larynx preservation. Consequently, multiple recent efforts have been made to integrate novel immunotherapies into the current treatment algorithm for LSCC. In particular, perioperative immunotherapy regimens appear to be the most promising approach for preserving laryngeal function and optimizing event-free and overall survival rates in the locally advanced setting. In the recurrent/metastatic setting, the 5-year overall survival rate is approximately 20% with pembrolizumab-based regimens. Primary and secondary resistance to immunotherapy is frequently observed in the majority of patients. Along with trials of checkpoint inhibitor monotherapy, combinatorial approaches with novel immunotherapies, bispecific antibodies, targeted therapies, and antibody-drug conjugates are being explored for the treatment of recurrent/metastatic laryngeal carcinoma. This article aims to discuss recent efforts to improve outcomes and quality of life for patients with locally advanced and recurrent/metastatic LSCC.

## Introduction

Laryngeal squamous cell carcinoma (LSCC) accounts for 180.000 cases and 100.000 deaths worldwide each year and predominantly affects males (1). A recent cohort study conducted in the United States suggests a decline in incidence, from 5.00 per 100.000 people (95% CI 4.70-5.32 per 100.000 people) to 2.26 per 100.000 people (95% CI 2.11-2.42 per 100.000 people) between 1986 and 2018, although mortality has not decreased at the same rate (2). Of note, the incidence rate in elderly patients above 65 years is approximately 3.3 times higher than in cases of adults between 35 and 64 years old (3). Single-modality treatment, such as radiotherapy (RT) alone or organ-preserving surgery, is the treatment of choice for early-stage T1 and T2 LSCC without nodal involvement. For locally advanced LSCC, current European Society of Medical Oncology (ESMO) Clinical Practice guidelines recommend a multimodality approach involving surgery and chemoradiation (CRT) (4). The treatment goals at this stage are to maximize survival while maintaining quality of life through organ-preserving strategies whenever possible. Unfortunately, recurrence occurs in approximately 50% of patients with human papillomavirus (HPV) negative locally advanced head and neck squamous cell carcinoma (HNSCC). In such cases, salvage surgery is preferred; however, for the majority of patients, palliative systemic therapy remains the only option.

**Figure 1:**
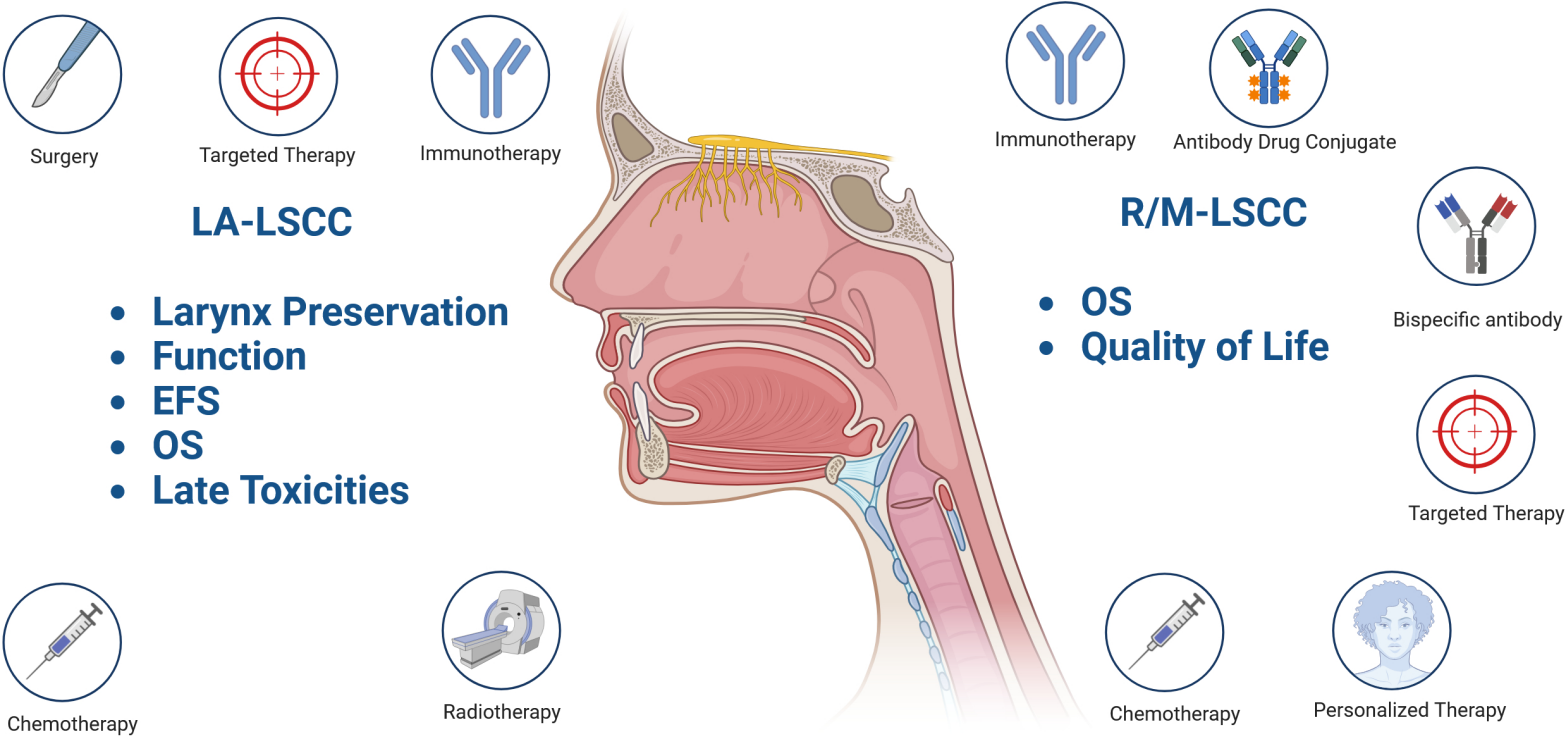
Treatment goals and current concepts explored in locally advanced (LA) and recurrent/metastatic (R/M) laryngeal carcinoma (LSCC). Event free survival (EFS); Overall survival (OS); “Created in BioRender. Fuereder, T. (2025) https://BioRender.com/c23r458”.

This review aims to discuss recent efforts to improve outcomes and quality of life for patients with locally advanced (LA) and recurrent/metastatic (R/M) LSCC (**Figure 1**).

## Recurrent and metastatic laryngeal cancer

There are a limited number of trials dedicated exclusively to R/M LSCC. Typically, R/M LSCC is included in clinical trials that focus on HNSCC, which also include oropharyngeal, oral cavity, and hypopharyngeal carcinomas. Given the diverse prognoses of the distinct subsites of HNSCC, it is not surprising that laryngeal cancer has a unique tumor microenvironment, characterized by a high degree of cellular heterogeneity, as demonstrated in recent single-cell transcriptomic analyses of metastatic laryngeal carcinoma (5). The trials presented below did not specifically report outcomes for LSCC patients, and the inclusion of both HPV-positive and HPV-negative tumors may lead to results that do not fully reflect the actual situation of laryngeal cancer. However, the subgroup analyses conducted do not indicate better or worse outcomes for LSCC patients compared to other HPV- negative subsites.

### Current status of systemic therapy

For over a decade, the EXTREME regimen (platinum/5-FU plus cetuximab) was the standard of care (SoC) for previously untreated R/M HNSCC. This regimen was established in a phase III trial comparing EXTREME to platinum/5-FU in 442 R/M HNSCC (111 LSCC) patients. EXTREME was superior to platinum/5-FU in terms of overall survival (OS: 10.1 months vs. 7.4 months; Hazard Ratio [HR]: 0.80, 95% Confidence Interval [CI] 0.64 to 0.99, p= 0.04) and progression-free survival (PFS: 5.6 months vs. 3.3 months; HR: 0.54, p< 0.001). Toxicity was comparable between the two arms, although grade 3/4 skin reactions and sepsis were more common in the EXTREME group (6).

More recently, the TPExtreme phase II trial compared cisplatin/docetaxel plus cetuximab (TPEx) to the EXTREME regimen in this population. 541 patients including 91 LSCC patients were randomized. While OS was similar between the two arms (14.5 months vs. 13.4 months; HR: 0.89, 95% CI 0.74 to 1.08, p= 0.23), the side effect profile was more beneficial in the TPEx arm. Grade ≥3 adverse events occurred in 81% of the TPEx group, while 93% in the EXTREME group (p<0.0001). The objective response rate (ORR) was comparable between the two groups (57% vs. 57%) (7).

The advent of immune-checkpoint inhibitors revolutionized the treatment of R/M HNSCC. In the first line setting of R/M HNSCC not amenable to curative local therapy the Keynote 048 phase III study defined a new standard of care. This study evaluated the efficacy of first line pembrolizumab (P) monotherapy and pembrolizumab plus chemotherapy (P+C) versus the EXTREME regimen in 882 R/M HNSCC patients of which 237 patients had R/M laryngeal cancer. The Keynote 048 trial demonstrated an OS benefit with P in the Programmed Cell Death 1 Ligand (PD-L1) combined positive score (CPS) ≥1 (OS 12.3 months vs. 10.3 months; HR: 0.78, 95% CI 0.64–0.96, p= 0.0089) and CPS ≥20 subgroups (OS 14.9 months vs. 10.7 months; HR: 0.61, 95% CI 0.45–0.83, p= 0.0007), and in combination with platinum/5-FU both in the total population (OS 13.0 months vs. 10.7 months; HR: 0.77, 95% CI 0.63–0.93, p= 0.0034) and the predefined CPS positive populations (CPS ≥1: OS 13.6 months vs. 10.4 months, HR: 0.65, 95% CI 0.53-0.8, p < 0.0001; CPS ≥20: OS 14.7 months vs. 11.0 months, HR: 0.65, 95% CI 0.45-0.82, p= 0.004) over the EXTREME regimen (8).

More recently a 5 year follow up analysis reported impressive long term survival results. The 5-year OS of P was 19.9% in the CPS ≥20 subgroup vs. 7.4 in the EXTREME arm. Likewise, the 5-year OS rate was longer in the P+C group vs. the comparator arm (CPS ≥20: 23.9% vs6.4%; CPS ≥1: 18.2% vs.4.3%) (9). The search for a predictive biomarker (beyond PD-L1 expression) to identify patients, who derive the maximum benefit from checkpoint inhibitor (CPI) therapy is still ongoing.

The single arm Phase IV Keynote B10 study recruited patients in the same setting, but patients were exposed to carboplatin/paclitaxel plus pembrolizumab. The primary endpoint was the overall response rate (ORR). 101 patients including 24 LSCC patients were treated. The ORR was 49% and 7% achieved a complete response (CR). The median OS was 13.1 months (95% CI 9.6 to 15.2) and comparable to what was reported in the Keynote 048 study. Serious treatment related adverse events occurred in 27% of the patients (10).

In platinum resistant patients the Keynote 040 and Checkmate 141 phase III trials led to the approval of pembrolizumab and nivolumab.

In the Keynote 040 trial 247 R/M HNSCC patients (including LSCC) were randomized to pembrolizumab or standard of care therapy (methotrexate 40-60 mg/m^2^, docetaxel 30-40 mg/m^2^ or cetuximab 250 mg/m^2^ weekly after a loading dose of 400 mg/m^2^). The median OS was 8.4 months with P and 6.9 months with standard of care (HR: 0.80, 95% CI 0.65–0.98; p=0.0161). The survival advantage for P was more pronounced in patients with a tumor proportion score (TPS) ≥50% (OS: 11.6 months vs. 6.6 months; HR: 0.53, 95% CI 0.35–0.81; p=0.014) (11).

The Checkmate 141 study had a similar trial design as the Keynote 040 trial comparing the efficacy of nivolumab with standard of care therapy in 361 platinum resistant R/M HNSCC patients including 49 patients with LSCC. The median OS was longer in nivolumab treated patients compared to SoC (methotrexate 40-60 mg/m^2^, docetaxel 75 mg/m^2^ or cetuximab 250 mg/m^2^ weekly following a loading dose of 400 mg/m^2^)(OS: 7.5 months vs. 5.1months; HR: 0.70, 97.73% CI 0.51–0.96; p=0.01) (12). The OS advantage was maintained in the 2-year follow-up analysis, demonstrating a 24-month OS rate of 16.9% for nivolumab compared to 6.0% in the SoC group (13).

**Table 1** summarizes the results of the pivotal CPI phase III trials conducted in R/M HNSCC.

**Table 1:** Pivotal phase III trials in R/M HNSCC investigating immune checkpoint inhibitors.

Trial Name (NCT)	Phase	Treatment	OS/PFS/DoR (months)	ORR/SD/PD
Keynote 048 (8) (NCT02358031)	III	Total Population Pembrolizumab Pembrolizumab plus Chemo Extreme PDL-1 CPS≥1 Pembrolizumab Pembrolizumab plus Chemo Extreme PDL-1 CPS≥20 Pembrolizumab Pembrolizumab plus Chemo Extreme	11.5/2.3/22.6 13.0/5.8/6.7 10.7/5.2/4.5 12.3/3.2/23.4 13.6/5.0/6.7 10.3/5.0/4.5 14.9/3.4/22.6 14.7/5.8/7.1 10.7/5.0/4.2	17%/27%/41% 36%/28%/17% 36%/34%/12% 19%/28%/39% 36%/26%/17% 35%/33%/13% 23%/30%/32% 43%/23%/15% 36%/35%/10%
Checkmate 651 (14) (NCT02741570)	III	Total Population Ipilimumab plus Nivolumab Extreme PDL-1 CPS≥1 Ipilimumab plus Nivolumab Extreme PDL-1 CPS≥20 Ipilimumab plus Nivolumab Extreme	13.9/3.3/16.6 13.5/6.7/5.9 15.9/4.2/ 13.2/6.1/ 17.6/5.4/32.6 14.6/7.0/7.0	24.2%/29.7%/30.1% 36.8%/36.4%/5.9% 27.6%/30.4%/26.8% 35.8%/36.3%/6.7% 34.1%/28.1%/23.2% 36.0%/31.5%/9.0%
Kestrel (15) (NCT02551159)	III	Total Population Durvalumab plus Tremelimumab Durvalumab Extreme PDL-1 high Durvalumab plus Tremelimumab Durvalumab Extreme	10.7/2.8/9.2 9.9/2.8/11.9 10.3/5.4/4.2 11.2/2.8/6.5 10.9/2.8/12.3 10.9/5.3/4.2.	21.8%/35.8%/39.0% 17.2%/35.8%/43.1% 49%/28.6%/13.6% 25.3%/32.1%/38.4% 16.2%/38.4%/40.4% 50%/28.7%/9.6%
Keynote 040 (11) (NCT02252042)	III	Total Population Pembrolizumab SoC PDL-1 TPS≥50% Pembrolizumab SoC	8.4/2.1/18.4 6.9/2.3/5.0 11.6/NR/Not reached 6.6/NR/6.9	14.6%/22.7%/43.7% 10.1%/26.2%/39.1% 26.6%/22.2%/40.7% 9.2%/23.1%/35.4%
Checkmate 141 (12, 13) (NCT02105636)	III	Total Population Nivolumab SoC	7.1/2.0/9.7 5.1/2.3/4.0	13.3%/22.9%/41.3% 5.8%/35.5%/34.7%
Eagle (16) (NCT02369874)	III	Total Population Durvalumab plus Tremelimumab Durvalumab SoC	6.5/2.0/7.4 7.6/2.1/12.9 8.3/3.7/3.7	18.2%/23.1%/55.9% 17.9/23.3%/54.6% 17.3/36.5%/34.5%

OS, Overall survival; PFS, Progression free survival; DOR, Duration of response; ORR, overall response rate; SD, stable disease; PD, progressive disease; PDL-1, Programmed cell death ligand-1; CPS, combined positive score; TPS, tumor proportion score; NR, not reported; Extreme platinum/5-FU plus cetuximab; SoC, standard of care.

### Immune checkpoint inhibitor combinatorial trials

In an attempt to further improve the efficacy of immunotherapy in R/M HNSCC, combinations of PD-(L)1 inhibitors with cytotoxic T-lymphocyte-associated protein 4 (CTLA-4) inhibitors were tested.

The Checkmate 651 trial recruited 947 patients, including 190 with LSCC. Patients were randomized to receive first-line ipilimumab plus nivolumab versus EXTREME. No statistically significant difference in OS was observed between the experimental and SoC groups (13.9 vs. 13.5 months; HR: 0.95, CI 97.9%, 0.80 to 1.13; p = 0.4951). Likewise, LSCC patients did not derive an OS benefit from ipilimumab/nivolumab (15.0 vs. 13.3 months; HR: 1.02). Of note, the duration of response (DoR) in the CPS ≥20 group was 32.6 months in the nivolumab/ipilimumab arm compared to 7.0 months in the SoC arm, which is longer than what was reported in the Keynote 048 study. Unfortunately, no biomarker was identified to select a subpopulation that might benefit from this regimen. Finally, it should be noted that the high rate of patients who received subsequent immunotherapy in the EXTREME arm (46.3%) may have contributed to the negative outcomes (14).

Similarly, the KESTREL phase III trial investigated the efficacy and safety of durvalumab (D) and durvalumab plus tremelimumab (DT) versus EXTREME as a first-line therapy for R/M HNSCC patients. A total of 823 patients were randomized, including 178 with LSCC. The study reported a similar OS in PD-L1 high patients, the primary endpoint, between the D, DT, and EXTREME groups (10.9 and 11.2 months versus 10.9 months; HR: 0.96, 95% CI, 0.69 to 1.32; p = 0.787; and HR: 1.05, 95% CI, 0.80 to 1.39) (15). However, an exploratory subgroup analysis suggested an OS benefit of DT versus EXTREME in patients with a blood tumor mutational burden (bTMB) ≥ 16 mutations/megabase (HR: 0.69, 95% CI, 0.39 to 1.25) (17).

The EAGLE trial investigated D vs. DT vs. SoC therapy (cetuximab, docetaxel, paclitaxel, methotrexate, 5-fluorouracil, tegafur/gimeracil/oteracil, or capecitabine, all dosed according to local regulations) in 736 platinum-resistant R/M HNSCC patients, of which 115 were LSCC patients. Again, no superiority of immunotherapy with D (HR: 0.88; 95% CI, 0.72 to 1.08; p = 0.2) or DT (HR: 1.04; 95% CI, 0.85 to 1.26; p = 0.76) over SoC could be demonstrated (16). A pooled analysis of the EAGLE and the phase II HAWK and CONDOR trials confirmed the potential role of bTMB as a biomarker in this setting. In EAGLE, an OS benefit with D and DT over EXTREME at an bTMB ≥ 16 mutations/megabase (HR: 0.39; 95% CI, 0.20 to 0.76 and HR: 0.38; 95% CI, 0.19 to 0.78) was demonstrated (18).

The LEAP-10 phase III trial evaluated the efficacy of P plus lenvatinib (an oral multikinase inhibitor that targets vascular endothelial growth factor (VEGF) receptors 1, 2, and 3; fibroblast growth factor (FGF) receptors 1, 2, 3, and 4; platelet-derived growth factor receptor α (PDFGRα), RET, and KIT) vs. P plus placebo in 511 previously untreated CPS ≥1 R/M HNSCC patients, including 111 LSCC patients. Although the combination of lenvatinib plus P showed a superior ORR compared to SoC (ORR 46.1% vs. 25.4%; p= 0.0000251), no difference in OS was observed (15.0 months vs. 17.9 months; HR: 1.19, 95% CI, 0.91 to 1.45, p= 0.8820) (19).

The INTERLINK-1 phase III study investigated the efficacy of cetuximab in combination with the NKG2A inhibitor monalizumab vs. cetuximab plus placebo in 624 human papilloma virus (HPV) unrelated R/M HNSCC patients, who were previously treated with a PD-(L)1 inhibitor and platinum chemotherapy. No difference in OS was observed between the groups (8.8 months vs. 8.6 months; HR: 1.00, 95% CI, 0.66 to 1.54) at the interim analysis and the trial was stopped as the futility criteria were met (20).

Since the aforementioned phase III trials did not meet their primary endpoints, novel strategies and biomarker-driven approaches appear to be necessary.

### Immunotherapy ongoing trials and novel approaches

The novel antibody BCA-101 is a bifunctional anti-epidermal growth factor receptor (EGFR) antibody linked to the extracellular domain of human transforming growth factor beta receptor II (TGFβRII). The efficacy and safety of this compound were evaluated in combination with P in a phase II study involving 42 previously untreated R/M HNSCC patients with PD-L1 CPS ≥1. The ORR in HPV-negative patients was promising, with an ORR of 54% in CPS 1-19 patients and 73% in CPS ≥20 patients. Three of the four patients with LSCC achieved a response. The median PFS was not reached and 57% of the patients had a PFS > 6 months. Toxicity was manageable, with the majority of patients (76%) developing skin rash (all grades). The most severe grade 3/4 treatment-related adverse events, occurring in 3 patients, included pericarditis, tracheal hemorrhage, and elevated blood alkaline phosphatase (21, 22). Based on these results, a phase III trial is planned.

Another bispecific antibody termed petosemtamab, which targets EGFR and Leucine-rich repeat-containing G-protein coupled receptor 5 (LGR5), demonstrated promising results in a phase II study. This study was conducted in 45 patients with PD-L1 CPS ≥1 HNSCC, including 7 with LSCC, and combined petosemtamab with P in the first-line setting. The ORR was 67% among 24 evaluable patients. The safety profile was manageable, with all patients experiencing dermatitis/skin rash. Grade 3/4 treatment-related adverse events occurred in 11 patients (24%) (23).

A single-center phase I/II study investigated the combination of the vascular endothelial growth factor receptor (VEGFR) inhibitor ramucirumab plus P in 40 R/M HNSCC patients, including 4 (11%) with LSCC. The ORR in the evaluable population was 55% (95% CI, 38% to 70%). The median PFS was 5.5 months, and the OS was 14.5 months. The side effect profile was manageable, and no treatment-related deaths were reported, although 2 patients experienced ramucirumab-related grade 4 events, including an infusion reaction and myocardial infarction-related ventricular tachycardia (24). The randomized Rambro2 trial is currently exploring this regimen (NCT05980000).

The efficacy and safety of P in combination with the receptor tyrosine kinase inhibitor (TKI) cabozantinib with activity against a broad range of targets, such as MET, RET, AXL, VEGFR2, FLT3, and c-KIT was evaluated in a phase II study in 36 R/M HNSCC (4 LSCC patients were enrolled). The ORR was 52% and the DCR 91%. The median OS and PFS were 22.3 months and 14.6 months respectively. 50% of the patients experienced a grade 3/4 adverse event and in 17% of the cases a grade 3/4 treatment related adverse event was reported. No treatment related death occurred (25).

This investigator-initiated Phase II trial provided the rationale to initiate the STELLAR-305 Phase II/III study investigating the efficacy of the TKI zanzalintinib in combination with P in PD-L1-positive R/M HNSCC patients (NCT06082167).

The TACTI-003 Phase IIb study evaluated the efficacy of first line P in combination with the soluble LAG-3 fusion protein eftilagimod alpha (EFTI) in CPS <1 and CPS ≥1 R/M HNSCC patients. In the CPS≥1 cohorts EFTI plus P was compared to P alone in 118 patients of which 35.5% were LSCC patients. The ORR, which was the primary endpoint, was 19% in the combination group and 16% in the P monotherapy arm and the disease control rate (DCR) was 72.4% vs. 63.3%. In the CPS ≥20 population the ORR was 31% vs. 18.5% and the DCR 75.9% vs. 59.3%. The DoR was 17.5 months and 17.1 months, respectively. In the CPS<1 population 31 patients (32.3% with LSCC) were included. The ORR was 35.5% and the DCR 58.1%. No new safety signals were reported for all 3 cohorts (26, 27). The results of this study are difficult to interpret due to the small sample size and the weak association between ORR and CPS score, even in the pembrolizumab monotherapy arm. Phase III studies are needed to confirm these findings. The novel anti-HGF antibody ficlatuzumab was investigated in a clinical trial, either alone or in combination with cetuximab, in platinum- and cetuximab-resistant R/M HNSCC patients, with prior immunotherapy exposure being mandatory. A total of 27 patients were enrolled in the ficlatuzumab monotherapy cohort, and 33 patients in the combination arm. Seven patients with LSCC were included. While the monotherapy arm was closed early due to futility, the combination arm reported promising results. Ficlatuzumab plus cetuximab therapy resulted in a PFS of 3.7 months, an OS of 7.4 months, and an ORR of 19% in this heavily pretreated population. Exploratory analysis suggested a more pronounced benefit in HPV-negative patients. The most common toxicities were skin rash, hypoalbuminemia, and edema (28). A phase III trial is currently planned to test this combination in a larger population (NCT06064877).

Three clinical studies demonstrated the efficacy of immune checkpoint inhibitors in combination with cetuximab.

Pembrolizumab plus cetuximab was investigated in a phase II trial in 33 platinum-resistant or ineligible patients of which 3 patients had LSCC. The ORR was 45% (95% CI 28% to 62%). Median PFS and OS were 6.5 months and 18.4 months, respectively. The most common grade 3-4 adverse event was oral mucositis (9%) (29).

Nivolumab plus cetuximab was tested in a similar designed study. 95 patients were enrolled in either cohort A, who were pre-treated, or cohort B, who were treatment-naïve. Both cohorts included 6 LSCC patients. The median OS and PFS in cohort A and B were 11.4 months, 3.4 months, 20.2 months and 6.15 months. The ORR were 22% in cohort A and 37% in cohort B. No new safety signals were reported (30).

Finally, durvalumab in combination with cetuximab showed similar results in 35 patients of which 31% were LSCC patients. Pretreatment with platinum therapy, immunotherapy and cetuximab was allowed. The ORR was 39%, the DoR 8.6 months, PFS 5.8 months and OS 9.6 months. Grade 3/4 treatment related AEs were reported in 34% of the patients and no treatment related death occurred (31).

Based on these studies, immunotherapy in combination cetuximab is a recommended option according to the NCCN guidelines for R/M HNSCC.

### Antibody–drug conjugates

Antibody-drug conjugates (ADCs) consist of a monoclonal antibody attached to a cytotoxic agent and belong to a novel category of immunoconjugates. Their mode of action includes binding to a specific antigen, being taken into the cell, and releasing the cytotoxic agent, which leads to the destruction of tumor cells. Furthermore, ADCs can also stimulate the immune system through various processes, such as antibody-dependent cellular cytotoxicity (ADCC) (32).

Tisotumab Vedotin, an ADC directed against tissue factor, was investigated in a phase II trial in platinum and immunotherapy pre-treated R/M HNSCC patients. A total of 40 patients were enrolled, including 13 LSCC/hypoharyngeal cancer patients. The ORR was 32.5% and the DCR 42.5%. The most common grade ≥ 3 treatment related adverse events of special interest were peripheral neuropathy (10.0%), ocular toxicity (5%) and bleeding (2.5%) (33).

Enfortumab Vedotin, an ADC directed against NECTIN-4, demonstrated activity in R/M HNSCC in a single arm phase II trial in 46 platinum and immunotherapy pre-treated HNSCC patients (including 10 LSCC patients). The ORR and DCR were 23.9% and 56.5%. The median DoR, PFS and OS were 9.4 months, 3.9 and 6.0 months, respectively. No new safety signals were reported (34).

The TROPiCS-03 trial evaluated the efficacy of the trophoblast antigen 2 (TROP-2) ADC Sacituzumab Govitecan in a phase II trial in 43 HNSSC patients, who progressed after platin-based chemotherapy and immunotherapy. 26% of the patients had LSCC. The ORR was 16% and the DCR 28%. A DoR of 4.2 months and a PFS of 4.1 months were reported. Grade ≥3 treatment related adverse events were observed in 44% of the patients (35).

Another novel ADC, termed Sigvotatug Vedotin, which targets Integrin beta 6 (ITGB6), was administered in a basket trial in 56 pre-treated R/M HNSCC patients. The ORR was 23.2% with a median DOR of 5.5 months. Fatigue, diarrhea, nausea and peripheral neuropathy were the most common treatment related adverse events (36).

Multiple early-stage clinical studies are currently ongoing, exploring the efficacy of various ADCs in HNSCC (recently comprehensively reviewed by Park et al.), though their role in HNSCC and LSCC is yet to be determined (37).

### Other strategies

Additional approaches to tackle HPV negative R/M HNSCC are currently under investigation including cancer vaccine studies or adoptive cell therapy trials. Those studies are at early stage and the clinical role unclear. A personalized approach employing molecular sequencing methods might be beneficial (38).

#### Key takeaways R/M HNSCC including LSCC

- Pembrolizumab, with or without platinum/5-FU, is the current standard of care for R/M LSCC with PD-L1 CPS ≥1.
- Combinations of CTLA-4 and PD-(L)1 inhibitors have failed to improve survival compared to cetuximab plus chemotherapy in phase III trials.
- Single-arm trials of CPI and cetuximab suggest an improved overall response rate.
- Bispecific antibodies may emerge as new treatment options, as they have shown encouraging response rates in phase II trials.
- Various antibody-drug conjugates targeting different therapeutic pathways have demonstrated signs of clinical activity in early trials.

## Locally advanced laryngeal squamous cell carcinoma

As stated above, total laryngectomy (TL) and neck dissection followed by adjuvant radiotherapy (RT) or concurrent chemoradiotherapy had been the primary therapeutic approach for many decades. While this approach resulted in encouraging cure rates, the morbidity associated with TL is significant, leading to detrimental effects on both functional and social aspects, ultimately impairing the quality of life (39–41). Consequently, clinical trials have focused on organ preservation strategies, incorporating systemic therapy, to improve functional outcome without compromising survival.

### Current status of non-surgical strategies for larynx preservation

Approximately 40 years ago, the first trials were published demonstrating high response rates with induction chemotherapy. The administration of doublet chemotherapy, consisting of cisplatin and fluorouracil (PF), prior to surgery showed significant clinical activity, with response rates of 85-90%. Complete responses were reported in 35-55% of study participants, with pathological confirmation achieved in approximately 50% of cases (42, 43). Moreover, response following induction therapy was associated with superior survival in subsequent analyses (44, 45). An early prospective single-arm trial suggested that the response to induction chemotherapy might predict radiosensitivity. This series revealed that among patients with chemotherapy-sensitive disease, subsequent RT led to a complete response (CR) rate of 97%. In contrast, for those malignancies that did not respond to cisplatin-based induction, the CR rate following RT was only 6% (46).

The Veterans´Administration Laryngeal Cancer Study Group (VALCSG) trial represents the first phase III trial comparing TL with a larynx preservation (LP) approach consisting of induction chemotherapy followed by RT. Between 1985 and 1988 332 patients with LA LSCC (T1-4, N0-3; T1N1 excluded) were randomized to TL and postoperative RT or the LP arm consisting of 3 cycles induction chemotherapy with PF followed by RT in case of response (defined as 50% reduction). Patients not achieving response were treated with TL and postoperative RT. In the LP arm response was achieved in 85% (PR 54%, CR 31%) of patients (47). After 3 years follow-up a LP rate of 62% was reported. However, in patients with T4 primaries, 56% of patients ultimately required laryngectomy, which was more frequently associated with the glottic subsite and gross cartilage invasion. Of note, long-term follow-up revealed comparable OS in both treatment arms (35% vs. 39% in the LP arm). Further analyses provided insights regarding the pattern of failure: Induction chemotherapy was associated with a higher rate of local recurrence but better outcome regarding distant metastasis (48).

A second phase III trial, conducted by the European Organisation for Research and Treatment of Cancer (EORTC 24991) compared induction chemotherapy followed by RT and TL followed by postoperative RT in patients with LA hypopharyngeal cancer (T2-4, N0-2). Of the 202 included patients, 97 patients received induction chemotherapy consisting of 3 cycles PF. Complete response and partial response (PR) were observed in 54% and 32% (ORR 86%), respectively. CR rates upon induction declined with increasing tumor stage with 82% in T2, 48% in T3 and 0% in T4. A larynx preservation rate of 64% was observed in the LP arm. Similar to the VALCSG trial, OS was comparable between the TL and LP (13.8% vs. 13.1%, respectively) arm (49, 50). Collectively, these two trials showed feasibility of a larynx preservation concept, consisting of induction chemotherapy followed by RT, without jeopardizing survival.

The following Radiation Therapy Oncology Group (RTOG) 91-11 trial compared concomitant cisplatin plus radiotherapy versus induction chemotherapy (PF) followed by definitive RT versus RT alone in patients with LA glottic (31%) and supraglottic (69%) laryngeal cancer. Patients with high volume T4 primaries (extensive supraglottic tumors with invasion deep into the tongue musculature and extensive cancers with tumor penetration through the cartilage) were excluded. At 2 years, the LP rate in patients receiving CRT (88%) was significantly superior compared to patients receiving induction chemotherapy (75%, p=0.005) or RT alone (70%, p<0.001) (51). Long-term analysis (10 years median follow-up) continued to show that LP rates were significantly higher in the CRT (81.7%) compared to the other treatment arms (HR: 0.58, 95% CI 0.37-0.89, p=0.005 for CRT vs. RT plus induction chemotherapy). Moreover, results indicate that the addition of induction chemotherapy does not have an impact on LP, as no significant difference regarding LP rates were observed between the RT alone (63.8%) and induction chemotherapy (67.5%) arm in long-term analysis. Regarding OS no significant difference at 5- and 10 years were observed for the three treatment arms. However, survival curves did separate after 4.5 years in favor of the subgroup receiving induction chemotherapy compared to the CRT subgroup. Patients receiving CRT showed the lowest rate of laryngeal cancer-related death compared to the other treatment arms but high rates of non-cancer-related deaths (30.8% vs. 20.8% with induction chemotherapy and16.9% with RT alone). This finding raises the question whether unrecognized long-term toxicities of CRT treatment have contributed to an increase of non-cancer-related mortality (52). No conclusive explanations have been provided so far, and therefore this matter remains elusive.

In an effort to explore the efficacy of a treatment concept consisting of alternating sequences of PF and RT compared to induction chemotherapy followed by RT the EORTC conducted a phase III trial (EORTC 24954) in patients with resectable stage III/IV laryngeal (34%) or hypopharyngeal (66%) cancer who required TL. In the sequential arm patients received alternating sequence of PF and a two week course of RT with 20 Gy (total of three courses). The induction chemotherapy arm consisted of two cycles PF with subsequent response evaluation. Substantial regression of tumor volume or at least partial recovery of larynx mobility was defined as partial response. Patients showing response upon two cycles PF underwent two additional cycles followed by RT (70Gy). Non-responders were treated by laryngectomy and postoperative RT. No significant difference was observed for the primary endpoint survival with a functional larynx (local control, no tracheotomy or feeding tube) or secondary endpoints including progression-free survival, overall survival or LP rates. A median survival time with a functional larynx of 2.3 years was reported for the alternating sequence arm compared to 1.6 years in the induction subgroup. Median overall survival was comparable with 5.0 and 5.1 years in the induction and alternating sequence arm, respectively. A numerical benefit for the alternating sequence group was observed regarding LP rates and laryngeal functioning but failed to reach statistical significance (53, 54). Since no significant improvement due to the alternating regimen was observed this treatment concept was not pursued.

Randomized trials evaluating the addition of docetaxel to induction PF (TPF) in head and neck cancer revealed increased efficacy of a triple chemotherapy induction compared to PF (55–57). In the GORTEC 2000-2001 study (larynx and hypopharynx cancer patients included) higher response rates were observed in the TPF arm compared to PF induction with 80% versus 59% (p=0.02), respectively. Higher response rates consequently translated into a higher number of patients undergoing definitive radiotherapy. Finally, LP rates after 3 years were significantly higher in the TPF arm (70%) compared to PF induction (57.5%; p=0.03). Overall survival (60% in each arm, p=0.57), local recurrence and late salvage surgery were comparable between study arms. Analysis according to primary site was not possible due to the small sample size (58).

The randomized phase II TREMPLIN study (radiotherapy with cisplatin versus radiotherapy with cetuximab after induction chemotherapy for larynx preservation) evaluated whether a treatment concept consisting of TPF induction followed by radiotherapy plus concurrent cisplatin or cetuximab would increase LP rates in patients with larynx or hypopharynx cancer (T2-3; N0-3). The trial failed to achieve the prespecified end point of an 80% LP rate three months after completion of treatment. This was attributed to a high dropout rate (24%) due to substantial toxicity caused by TPF and insufficient response rates upon induction. Additionally, cetuximab proved to be at least as toxic as cisplatin when given as a radiosensitizer and was associated with a lower local control rate. Of note, LP rate in this trial was not increased when compared to the GORTEC 2000-2001 study (59).

Based on the above-mentioned trials, current guidelines from the ESMO advocate the use of LP concepts in the treatment of LA laryngeal and hypopharyngeal squamous cell carcinoma (4).

For laryngeal carcinoma staged as cT3b N0-N3 or T1-2 N2-N3, the primary management approach is concurrent CRT or organ-preserving surgery (4). In cases necessitating TL, treatment, options include CRT or induction chemotherapy followed by RT in responders (complete or partial). Persistent stable disease (SD) or progressive disease post-induction warrants surgical intervention with TL and neck dissection.

In T4a laryngeal carcinoma, the standard of care is TL with neck dissection, followed by adjuvant RT or CRT. For patients with contraindications or refusing surgery, options include CRT or induction chemotherapy with subsequent management contingent on treatment response. Enrolment in organ preservation clinical trials may also be considered.

In hypopharyngeal carcinoma (cT1-3, any N) who are candidates for total laryngopharyngectomy, three primary treatment approaches are suggested: induction chemotherapy followed by subsequent treatment based on response and risk factors; definitive CRT or TL with neck dissection and adjuvant RT or CRT according to pathological findings.

### Future directions

Current guidelines indicate that the optimal approach for larynx preservation strategies in patients requiring TL cannot be determined based on the available evidence. As a result, the debate over the best treatment protocol—one that balances survival, locoregional control, and acceptable late toxicity with good functional outcomes—remains unresolved. In particular, how to increase the frequency of larynx preservation without compromising overall survival continues to be an area of active research. In addressing these challenges, data from the aforementioned RTOG 91-11 trial has provided key insights. This trial compared different treatment protocols for LA LSCC. A re-analysis performed by Licitra et al. suggested that induction chemotherapy followed by RT could be the most effective strategy for achieving LP (60). The re-analysis argues that the trial does not demonstrate any clear clinical superiority of CRT over IC followed by RT in terms of larynx preservation, suggesting that sequential treatment with IC plus RT may achieve better long-term survival rates for patients with or without the larynx.

However, it is important to note that the induction chemotherapy regimen used in RTOG 91-11 (PF) is no longer considered as the most effective combination for induction treatment. Current protocols favor taxane-based regimens, such as TPF, which are associated with improved outcome. It is anticipated that the use of TPF-based IC could offer further benefits, potentially enhancing LP rates and long-term survival without worsening toxicity or functional outcomes.

As such, the GORTEC group developed the SALTORL trial (NCT03340896): In this study, patients with T2-3, N 0-2 larynx or hypopharynx carcinoma not amendable for partial surgery have been included. In a randomized approach, this study aimed to compare three cycles of induction TPF followed by subsequent therapy according to response with CRT using high dose cisplatin. Laryngoesophageal dysfunction free survival was the primary endpoint. Although enrollment in the trial has concluded, results have not yet been reported.

### Immune checkpoint inhibitors plus radiotherapy

Based on the approval of immune checkpoint inhibitors in R/M HNSCC, phase III studies evaluated those agents in the locally advanced setting in combination with CRT. The JAVELIN Head and Neck 100 and KEYNOTE-412 trials investigated whether the addition of a checkpoint inhibitor during concurrent radiochemotherapy with high dose cisplatin followed by maintenance immunotherapy might improve survival in LA HNSCC (61, 62). Both trials failed to meet the primary endpoint that was defined as improvement of event-free survival (EFS) compared to standard of care plus placebo.

In the Keynote 412 trial 804 patients were randomized including 178 LSCC patients. The EFS for pembrolizumab plus CRT was not reached and 46.6 months in the CRT arm (HR: 0.83, 95% CI 0.68 to 1.03; p=0.043; significance threshold: p ≤ 0.024) (62).

Similarly, the JAVELIN Head and Neck 100 trial enrolled 697 patients, including 124 with LSCC, and compared avelumab plus CRT versus CRT alone. Median PFS was not reached (95% CI 16.9 months–not estimable) in the avelumab group and not reached (2.0 months–not estimable) in the CRT group (HR: 1.21, 95% CI 0.93 to 1.57; p=0·92) (61).

In cisplatin-unfit patients the addition of avelumab to cetuximab during concurrent radiochemotherapy did not demonstrate a PFS benefit compared to cetuximab (PFS at 2years: 44% vs. 31%; HR: 0.84, 95% CI 0.62-1.15;p= 0.14) in the initial analysis of the phase III GORTEC-REACH trial (63). However, in a longer follow up the PFS curves separated in favor of avelumab-cetuximab RT (PFS at 4 years: 33.7% vs. 18.4%; HR: 0.80, 95% CI 0.60 to 1.06;p= 0.059), although no OS benefit was observed (HR: 1.05, 95% CI 0.76 to 1.43;p= 0.77) (64).

A randomized phase II trial emphasized on the optimal timing of checkpoint inhibitor (pembrolizumab) treatment in patients undergoing concurrent high-dose cisplatin radiochemotherapy. This study found a numerical improvement of PFS (67% vs. 49% at 4 years; HR: 0.57, 95% CI 0.26 to 1.28;p= 0.17) and OS (83% vs. 71% at 4 years; HR: 0.51, 95% CI 0.19 to 1.37;p= 0.18) in patients receiving sequential pembrolizumab after concurrent radiochemotherapy compared to patients receiving checkpoint inhibitor therapy during concurrent radiochemotherapy (65).

However, the IMvoke010 phase III study, investigating atezolizumab after definitive treatment in 406 LA-HNSCC including 70 LSCC patients, did not show a benefit for sequential checkpoint inhibition. The 2-year EFS was 59.5 months for atezolizumab vs. 52.7 months for placebo (HR: 0.94, 95% CI 0.70 to 1.26; p= 0.68) (66).

The Nivo-Postop trial (NCT03576417) is a phase III randomized study evaluating the efficacy of postoperative nivolumab in combination with cisplatin-based radiochemotherapy compared to CRT alone in patients with high-risk, resected HNSCC. The study showed a statistically significant improvement in DFS according to a recent press release (67).

### Neoadjuvant immune checkpoint inhibitor trials

Collectively, trials investigating the incorporation of checkpoint inhibitors during or after concurrent chemoradiotherapy in LA-HNSCC have yielded disappointing results thus far. The concurrent administration of high-dose radiation to lymphatic chains may impair T-cell priming and cross-presentation in the lymph nodes, potentially reducing the effectiveness of checkpoint inhibitors in this treatment setting. From this perspective, checkpoint inhibitor treatment prior to definitive treatment might be a more appealing approach. Multiple phase II trials evaluated the efficacy of neoadjuvant immunotherapy in LA-HNSCC:

Wise-Draper et al. reported a phase 2 trial conducted in 92 intermediate and high-risk LA-HNSCC (10 LSCC) patients, who were treated with neoadjuvant and adjuvant pembrolizumab. One-year DFS was 97% in the intermediate-risk group and 66% in the high-risk group. In total 7% of the patients achieved a major pathological response (mPR; ≥90% tumor necrosis) (68).

Uppaluri et al. reported on another phase II study in which 36 LA HNSCC (including 10 LSCC) patientsreceived one cycle of neoadjuvant pembrolizumab (cohort 1). Pathological tumor response ≥50% occurred in 22% of these patients and no surgical delays were observed (69). In cohort 2, 29 additional patients were treated with two doses of pembrolizumab. In 25 evaluable patients the pathological tumor response ≥50% increased to 44% (70).

The phase III KEYNOTE-689 trial compared neoadjuvant pembrolizumab, followed after surgery by adjuvant pembrolizumab plus radiotherapy with or without cisplatin (depending on risk factors), and maintenance pembrolizumab versus adjuvant radiotherapy with or without cisplatin alone in patients with newly diagnosed resectable stage III or IVA LA HNSCC. At a pre-specified first interim analysis there was a statistically significant improvement in EFS (the primary endpoint) for patients in the pembrolizumab arm. The study also showed a statistically significant improvement in mPR according to a recent press release (71). Final data on any survival benefit have to be awaited.

Finally, dual checkpoint inhibitor phase II studies such as the IMCISION trial evaluated the efficacy of nivolumab plus ipilimumab in 26 LA-HNSCC patients. The mPR rate was 35% and no safety issues such as surgical delays were reported (72).

Trials focusing on locally advanced laryngeal and hypopharyngeal cancer have been conducted to explore the use of checkpoint inhibitors within an induction chemotherapy approach.

In a single arm phase II trial conducted in China, three cycles of induction chemotherapy (cisplatin/paclitaxel) combined with toripalimab were tested in resectable LA laryngeal/hypopharyngeal squamous cell carcinoma. In patients achieving CR or PR treatment was followed by cisplatin-based CRT and subsequent toripalimab consolidation over 8 cycles. Primary endpoint was LP rate 3 months after completion of RT. Of 27 enrolled patients (18 hypopharynx; 9 larynx), 26 patients completed induction chemo-immunotherapy and CR/PR was observed in 21 patients. At 3 months post CRT, LP rate was 88.9%. No clear association of PD-L1 CPS and ORR, LP rate, or PFS was observed. Of note, five patients underwent pre-treatment tracheostomy due to impaired laryngeal function. In a *post-hoc* analysis impaired larynx function with the need of pre-treatment tracheostomy was associated with inferior survival (73).

This finding might suggest that highly infiltrative and extensive tumors may not be suitable candidates for induction chemo-immunotherapy and may be better treated with upfront total laryngectomy.

Another phase II single center trial, including locally advanced hypopharyngeal squamous cell carcinoma patients, aimed to determine the efficacy of an induction immune-chemotherapy consisting of three cycles cisplatin/docetaxel/capectabine and camrelizumab. In case of response (PR or CR), patients received RT combined with camrelizumab and followed by camrelizumab maintenance for up to 18 cycles. ORR after induction was defined as primary endpoint. In the 51 enrolled patients, ORR was 82.4%. At a median follow up of 23.7 months, two-year OS rate, PFS rate and LP rate were 83.0%, 77.1% and 70%, respectively. Exploratory analyses revealed no association of PD-L1 expression and response. Interestingly, ten patients underwent surgical treatment (SD upon induction n=6; suspected progression n=3, relapse of tuberculosis during radiotherapy n=1). Four of those patients displayed pathological complete response, highlighting potential discrepancies between radiological and pathological response assessment (74).

In an attempt to reduce treatment-related morbidity in LA LSCC, Ferrarotto and colleagues conducted the ICoLP trial aiming to achieve LP with a chemo-immunotherapy approach without additional local treatments. Stage II-III laryngeal carcinoma patients were treated with two cycles induction immuno-chemotherapy (pembrolizumab/platinum/docetaxel) with subsequent restaging by laryngoscopy and imaging. In case of progressive disease, patients received local therapy. Patients achieving at least stable disease received two additional immuno-chemotherapy cycles and were thereafter restaged by imaging and biopsy. Patients, in whom a pathological complete remission (pCR) was observed, received four cycles of pembrolizumab consolidation. In the absence of pCR, local treatment was applied. Co-primary endpoints were disease control rate (DCR) after 2 cycles and pCR rate after 4 cycles. In the 24 treated patients, no disease progression (CR n=5; PR n=12; SD n=6) was observed and therefore achieving a DCR of 100%. A pCR after 4 cycles immuno-chemotherapy was detected in 18 patients (78.3%). During follow-up, recurrence of laryngeal cancer was observed in 7 of 18 patients (38.9%) with pCR. All of them received salvage therapy (radiochemotherapy n=5; radiotherapy n=1; TL n=1). It is noteworthy that the majority of recurrences occurred within 5 months after end of treatment. Within a median follow-up of 20.4 months, the LPR of the ICoLP treatment concept was 91.7% (75).

These trials suggest high efficacy of an-upfront systemic treatment approach using checkpoint-inhibitors with a chemotherapy backbone that might lead to improved LPR rates and survival. However, current evidence seems to be rather hypothesis generating so far, but serves as a rationale for future randomized trials.

In this context the European Larynx Preservation Study (ELOS; NCT06137378) will explore the efficacy of the addition of pembrolizumab to induction chemotherapy (docetaxel/cisplatin) in patients with locally advanced stage III or IVa/b laryngeal or hypopharyngeal squamous cell carcinoma with PD-L1 expression (CPS ≥ 1) in a randomized approach. After the first chemotherapy +/- pembrolizumab cycle, patients with ≥30% tumor shrinkage continue with two additional cycles induction chemotherapy +/- pembrolizumab followed by radiotherapy. Non-responders undergo total laryngectomy. In the experimental arm, pembrolizumab is administered regardless of tumor response over a duration of 12 months, aiming to improve laryngectomy-free survival (76).

**Table 2** summarizes ongoing phase II/III immunotherapy trials evaluating either laryngeal preservation and/or neoadjuvant strategies.

**Table 2:** Selected future trials in LA-LSCC and HNSCC investigating neoadjuvant or laryngeal preservation strategies.

Trial Name (NCT)	Phase	Setting	Compound	Primary Endpoint
NCT04943445	II	LA-LSCC	Pembrolizumab Carboplatin/Paclitaxel	Laryngectomy-free survival
NCT06554028	II	LA-LSCC	Tiselizumab Cisplatin/Docetaxel	Laryngeal preservation rate at 3 months after radiotherapy
NCT06611137	II	LA-LSCC	Toripalimab Cisplatin/Docetaxel SBRT	Objective response rate
NCT06039631	II	LA-LSCC	Toripalimab	PFS rate
NCT06137378	II	LA-LSCC	Pembrolizumab	Laryngectomy-free survival
NCT03700905	III	LA-HNSCC	Nivolumab Ipilimumab	DFS
NCT06102395	III	LA-HNSCC	Pembrolizumab Platinum/Taxane	pCR
NCT06647563	III	LA-HNSCC	Toripalimab Cetuximab Platinum/Taxane	EFS rate at 2-years
NCT05980715	III	LA-HNSCC after pCR	PD-1 inhibitor	OS, Grade 3-4 AEs
NCT03765918	III	LA-HNSCC	Pembrolizumab	EFS
NCT05582265	III	LA-HNSCC	Tiselizumab Cisplatin/Nab-Paclitaxel	EFS

EFS, Event free survival; OS, Overall survival; PFS, Progression free survival; DFS, Disease free survival; pCR, pathological complete response; LA, locally advanced; HNSCC, head and neck squamous cell carcinoma; LSCC, laryngeal squamous cell carcinoma; SBRT, stereotactic body radiation therapy.

Key takeaways in LA LSCC.

- Larynx preservation is a primary goal in locally advanced LSCC.
- Induction chemotherapy followed by radiotherapy or concurrent radiochemotherapy represents the current larynx preservation strategies for LA LSCC amenable to total laryngectomy.
- The addition of PD-(L)1 inhibitors to definitive radio(chemotherapy) has not improved overall survival in phase III trials.
- Several phase II trials suggest increased therapeutic efficacy with the incorporation of checkpoint inhibitors in neoadjuvant or induction therapy concepts.

## Conclusion

The optimal approach for treating locally advanced and recurrent/metastatic laryngeal squamous cell carcinoma remains a clinical challenge. In the LA setting, the primary treatment goal is to optimize larynx preservation rates while improving disease-free survival and overall survival, all while minimizing late toxicities. At present, for that purpose the neoadjuvant use of immunotherapy with or without chemotherapy appears to be the most promising approach, as the concurrent use of immunotherapy and radiotherapy thus far produced disappointing results. For R/M LSCC, the efficacy of immunotherapy remains limited in the absence of a predictive biomarker besides PD-L1 expression. Novel compounds, such as bispecific antibodies or antibody-drug conjugates, may improve outcomes in this setting, pending positive results from future trials.
